# Investigation of *Pseudomonas fluorescens* strain 3JW1 on preventing and reducing aflatoxin contaminations in peanuts

**DOI:** 10.1371/journal.pone.0178810

**Published:** 2017-06-22

**Authors:** Xiaona Yang, Qi Zhang, Zhi-Yuan Chen, Hongxia Liu, Peiwu Li

**Affiliations:** 1 Department of Plant Pathology, College of Plant Protection, Nanjing Agricultural University, Nanjing, P. R. China; 2 Key Laboratory of Integrated Management of Crop Diseases and Pests (Nanjing Agricultural University), Ministry of Education, Nanjing, P. R. China; 3 Oil Crops Research Institute of the Chinese Academy of Agricultural Sciences, Wuhan, P. R. China; 4 Key Laboratory of Biology and Genetic Improvement of Oil Crops, Ministry of Agriculture, Wuhan, P. R. China; 5 Key Laboratory of Detection for Mycotoxins, Ministry of Agriculture, Wuhan, P. R. China; 6 Laboratory of Risk Assessment for Oilseeds Products (Wuhan), Ministry of Agriculture, Wuhan, P. R. China; 7 Department of Plant Pathology and Crop Physiology, Louisiana State University Agricultural Center, Baton Rouge, Louisiana, United States of America; Agricultural University of Athens, GREECE

## Abstract

*Pseudomonas fluorescens* strain 3JW1, which has a broad-spectrum antimicrobial activity, was studied to investigate whether it affects the amounts of aflatoxin B_1_ (AFB_1_) produced by *Aspergillus flavus*. It was found that the bacterium reduced the amounts of AFB_1_ in potato dextrose broth (PDB) and peanut medium by 97.8% and 99.4%, respectively. It also reduced AFB_1_ by ~183 μg/kg (55.8%) when applied onto peanut kernels. This strain reduced AFB_1_ via three mechanisms. First, it significantly inhibited *A*. *flavus* growth; second, our data showed that strain 3JW1 inhibits aflatoxin biosynthesis by *A*. *flavus*; and third, *P*. *fluorescens* strain 3JW1 is capable of degrading AFB_1_ at a rate as high as 88.3% in 96 hours. This is the first report demonstrating that *Pseudomonas fluorescens* can reduce toxin contamination caused by *A*. *flavus* on peanut kernels. Our findings indicate that *P*. *fluorescens* strain 3JW1 had multiple effects including reducing *A*. *flavus* infection and aflatoxin contamination. And the results also highlight the potential applications of the strain 3JW1 for the biological control of aflatoxin contamination in peanuts and other susceptible crops.

## Introduction

*Aspergillus flavus* belongs to *Aspergillus* Section *Flavi*. The fungus is a common saprophyte that can infect susceptible crops, such as peanut, corn, cotton seeds and tree nuts under favorable conditions [1, 2]. The most hazardous effect of this type of fungi is the production of aflatoxins, including aflatoxin AFB_1_, AFB_2_, AFG_1_ and AFG_2_, a group of secondary metabolites produced mainly by *A*. *flavus*, *A*. *parasiticus* and several other species including *A*. *nomius*, *A*. *pseudotamarii*, *A*. *parvisclerotigenus*, and *A*. *bombycis* of section Flavi, *A*. *ochraceoroseus* and *A*. *rambellii* from section Ochraceorosei and *Emericella astellata* and *E*. *venezuelensis* from Nidulatans section [2–4]. AFB_1_ is regarded as the most carcinogenic biotoxin in nature [2–4]. Its toxicity is 10 times greater than that of potassium cyanide and 68 times greater than that of arsenic [5, 6]. This toxin has been shown to induce mutations, suppress immune function, reduce growth, increase human and animal liver diseases, promote cancer development and cause acute aflatoxicosis and even death [5–11]. Therefore, AFB_1_ has been classified as a class I human carcinogen by the International Agency for Research on Cancer (IARC) [8, 12]. As a result, many countries have enacted strict standards on allowable levels of aflatoxins in food and feed. For example, European Union regulations state that AFB_1_ content in crops shall not be greater than 2 μg/kg and that the total toxin content cannot be more than 4 μg/kg [13]. Thus, aflatoxin-contamination of food and feed not only poses serious health concerns, but also causes significant economic losses to farmers.

Peanuts (*Arachis hypogaea*) are rich in protein content and are considered a nutritional food for both animals and humans. In addition, they are a vital oilseed and food crop utilized in most areas of the world. However, peanuts are usually threatened by pre-harvest infection of *A*. *flavus* and *A*. *parasiticus* when their fruits are tender or wounded and encounter rainy days at about 22–35°C and by inadequate storage conditions with relatively high moisture (over 85%) and temperature (over 22°C), which are often two of the main contributing factors that lead to moldy peanuts, reduced seed viability and increased seed rot [11, 14].

For these reasons, determining how to effectively reduce *A*. *flavus* infection and subsequent aflatoxin contaminaiton has extremely important theoretical and practical significance. Currently, some measures have been taken to reduce the infection, including physical and chemical methods, such as good field management and pest control [15, 16]. However, the control of *A*. *flavus* and aflatoxin remains a global problem due to lack of effective control measures.

Recently, advances in green, environmental and health technologies have inspired renewed efforts to develop biological control strategies to reduce *A*. *flavus* infection and aflatoxin contamination. One of the major breakthroughs is the use of atoxigenic strains of *A*. *flavus* to compete with toxigenic *A*. *flavus* in the field, which has been shown to successfully reduce aflatoxin contamination in the U.S. and African countries [17–21]. Atehnheng *et al*. evaluated the abilities of eleven naturally occurring atoxigenic isolates in Nigeria to reduce aflatoxin contamination in corn in field studies during the 2005 and 2006 growing seasons and found relative levels of aflatoxin B_1_ + B_2_ reduction ranged from 70.1% to 99.9% [22].

However, the presence of partial toxin pathway gene cluster in the atoxigenic biocontrol strains is of special concern considering the recent reporting of possible recombination of *A*. *flavus* under natural conditions [23–24]. Therefore, the use of other antagonists in biocontrol of *A*. *flavus* has also been explored. For example, Palumbo *et al*. [25] found that strains of *Pseudomonas chlororaphis* and *P*. *fluorescens* from Mississippi maize field soil and maize rhizosphere samples could inhibit *A*. *flavus* growth in different media (i.e., liquid or agar media). Moreover, a strain of *Bacillus pumilus* isolated from Korean soybean sauce exhibited strong antifungal activity against the aflatoxin-producing fungi *A*. *flavus* and *A*. *parasiticus* [26]. A biocontrol yeast, *Pichia anomala* strain WRL-076, inhibited *A*. *flavus* spore germination and aflatoxin production [27]. Sangmanee and Hongpattarakere [28] reported that *Lactobacillus plantarum* K35 isolated from traditional Thai fermented rice noodles could effectively inhibit the growth and aflatoxin production of *A*. *flavus* TISTR304 and *A*. *parasiticus* TISTR3276.

The present study aimed to investigate the efficacy of a previously isolated *Pseudomonas fluorescens* strain 3JW1 in reducing AFB_1_ produced by *A*. *flavus*, and to explore the potential of applying *P*. *fluorescens* strain 3JW1 to reduce AFB_1_ in peanuts. *P*. *fluorescens* strain 3JW1, a non-pathogenic endophyte, was originally isolated from the stem of ginger and used safely to inhibit plant disease [29]. But there is no report of its efficacy against *Aspergillus flavus*. Therefore, how strain 3JW1 affects *A*. *flavus* growth and the subsequent aflatoxin production of the recovered *A*. *flavus* after being treated with the biocontrol agent were examined. In addition, the efficacy of this strain in suppressing aflatoxin contamination in peanut and on degrading AFB_1_ was also investigated.

## Materials and methods

### Materials

#### Strains

*Aspergillus flavus* strain 73 was isolated from an aflatoxin-contaminated peanut sample that was provided by the Oil Crops Research Institute of the Chinese Academy of Agricultural Sciences. *Pseudomonas fluorescens* 3JW1 was provided by the Biological Pesticides and Green Plant Protection Laboratory of Nanjing Agricultural University.

#### Media

Luria-Bertani (LB) medium (yeast extract 5 g/L, tryptone 10 g/L, NaCl 10 g/L, pH 7.0–7.2; autoclaved for 20 min at 121°C), Czapek medium (sodium nitrate 3 g/L, dipotassium hydrogen phosphate 1 g/L, magnesium sulphate 0.5 g/L, potassium chloride 0.5 g/L, ferrous sulfate 0.01 g/L, sucrose 30 g/L, and agar 20 g/L; autoclaved for 20 min at 121°C), Potato Dextrose Agar (PDA) medium (potato 200 g/L, dextrose 20 g/L, and agar 15 g/L; sterilized for 20 min at 121°C), Potato Dextrose Broth (PDB) medium (potato 200 g/L and dextrose 20 g/L; autoclaved for 20 min at 121°C) and peanut medium (potato 200 g/L, dextrose 20 g/L, and peanut flour 1.5 g/L; autoclaved for 20 minutes at 121°C) were prepared in house.

### Methods

#### Preparation of the biocontrol strain

*Pseudomonas fluorescens* strain 3JW1 was grown in LB medium for 24 h at 28°C with shaking at 200 r/min. *Escherichia coli* TOP10F′was grown in LB medium for 24 h at 37°C with shaking at 200 r/min. Cells were counted using a plate counting method.

#### Preparation of an *A*. *flavus* spore suspension

*A*. *flavus* spore suspensions were prepared by flooding 10-day-old cultures of *A*. *flavus* on PDB with sterile distilled water (containing 0.1% Tween 80) in a biosafety hood. Spores were counted using a hemocytometer [30].

#### Effect of *P*. *fluorescens* strain 3JW1 on fungal AFB_1_ production in PDB

*A*. *flavus* was mixed with strain 3JW1 in a 100-ml flask containing 15 ml of PDB. The final concentrations of *A*. *flavus* and the biocontrol strain were 5×10^5^ spores/ml and 1×10^7^ colony-forming units (CFU)/ml according to the previous study [30]. Control treatments include *A*. *flavus* alone in PDB and *A*. *flavus* mixed with 1×10^7^ CFU/ml *E*. *coli*. After 4 d (96 h) of incubation with constant shaking (200 r/min) at 28°C in an incubator shaker, the medium filtrate was collected (except mycelium), and the amount of AFB_1_ was determined by immune affinity column-high performance liquid chromatography (IAC-HPLC) [31]. In order to further determine whether any reduction on AFB_1_ was due to inhibition on fungal growth, *A*. *flavus* mycelium from PDB medium alone, PDB medium containing strain 3JW1 and PDB medium containing *E*. *coli* was harvested by filtration, followed by washing and drying at 80°C for 24 h. The mycelium dry weight obtained was recorded and compared. This study was repeated 3 times, each with 3 replications.

#### Effects of *P*. *fluorescens* strain 3JW1 on AFB_1_ production in peanut medium

In order to identify whether strain 3JW1 could play the same role in PDB with peanut powder, *A*. *flavus* was mixed with strain 3JW1 in a 100-ml flask containing 15 ml of peanut medium. The final concentrations of *A*. *flavus* and the biocontrol strain were 5×10^5^ spores/ml and 1×10^7^ colony-forming units (CFU)/ml, respectively. A control treatment with *A*. *flavus* in peanut medium alone and another control treatment of *A*. *flavus* mixed with *E*. *coli* at a final concentration of 1×10^7^ CFU/ml were also included. After 96 h of incubation with constant shaking (200 r/min) at 28°C in an incubator shaker, the medium filtrates were collected, and the amount of AFB_1_ was determined by immune affinity column-high performance liquid chromatography (IAC-HPLC). This study was repeated 3 times, each with 3 replications.

#### Efficacy of *P*. *fluorescens* strain 3JW1 in reducing AFB_1_ contamination in peanuts

In our preliminary efficacy study, 20 peanut kernels were used in each assay. Peanut kernels were surfaced-disinfected with 0.1% sodium hypochlorite for 1 min, rinsed with sterile distilled water 3 times for 30 second each time, and then air-dried in a petri dish. One ml of biocontrol strain at 1.0×10^7^ CFU/ml was added to the petri dish, and peanut kernels were dipped into petri dish containing the bacteria and shaken for 5 min to allow the bacteria to be absorbed by the peanuts. Then, 1 ml of *A*. *flavus* inoculum at 5×10^5^ spores/ml was added to each petri dish four hours later and the dishes were then shaken as above for 5 min. All petri dishes were then placed in an artificial weather chamber to maintain high humidity (85%) and were incubated at 28°C [30]. The amount of AFB_1_ was determined by IAC-HPLC 7 days later. Each treatment was replicated three times and this study was repeated four times.

#### The ability of *P*. *fluorescens* strain 3JW1 in suppressing aflatoxin biosynthesis or degrading aflatoxins

To determine whether the reduced aflatoxin production by the biocontrol strain was due to its inhibitory effect on aflatoxin biosynthesis or due to degradation of the produced aflatoxins by the biocontrol agent, the following two studies were conducted. For the first study, 100 μl of *A*. *flavus* and strain 3JW1 mixed culture was collected at the end of 96 h co-incubation on PDB and plated on petri dishes containing PDA medium. After 4 days, a single colony of *A*. *flavus* was transferred onto a new PDA plate. Conidia of *A*. *flavus* on the new plate were collected 14 days later, and were used to inoculate into 15 ml of PDB in a 100-ml flask at a final concentration of 5×10^5^ spores/ml. After 96 h of constantly shaking (200 r/min) the above culture at 28°C in an incubator shaker, the medium filtrate was collected and the amount of AFB_1_ was determined by IAC-HPLC. This study was conducted 3 times and each treatment was replicated three times.

For the second study, a 0.8 ml of 24 h old culture of strain 3JW1 in LB was added to a 1.5 ml microcentrifuge tube containing 0.2 ml of AFB_1_ standards (250 ng/ml). The tube was incubated on a shaker with constant shaking at 200 r/min and 28°C for 96 h at°Cbefore its AFB_1_ level was analyzed by IAC-HPLC. The fresh sterile LB and *E*. *coli* culture were included as controls, in which 0.8 ml of one day old *E*. *coli* TOP10F′ culture in LB or only LB was added to a 1.5 ml microcentrifuge tube containing 0.2 ml of AFB_1_ standards (250 ng/ml). The degradation rate was calculated using the formula [y = (x_1_ –x_2_) / x_1_×100%] [32]. Here, x_1_ represents the contents of AFB_1_ in the control treatment, x_2_ represents the contents of AFB_1_ in the treated group, and y represents the detoxification ratio. These studies were conducted 3 times and each treatment was replicated three times.

#### Data analysis

All data were analyzed for statistical significance by the least significant difference (LSD) test (p < 0.05) using the Data Processing System (DPS version 7.05; Hangzhou Rui Feng Information Technology Inc., Hangzhou, Zhejiang, China) statistical software package.

## Results and discussions

### Effects of the 3JW1 biocontrol strain on reducing AFB_1_ levels

#### Effects of *P*. *fluorescens* strain 3JW1 on AFB_1_ levels in PDB

**Fig 1:**
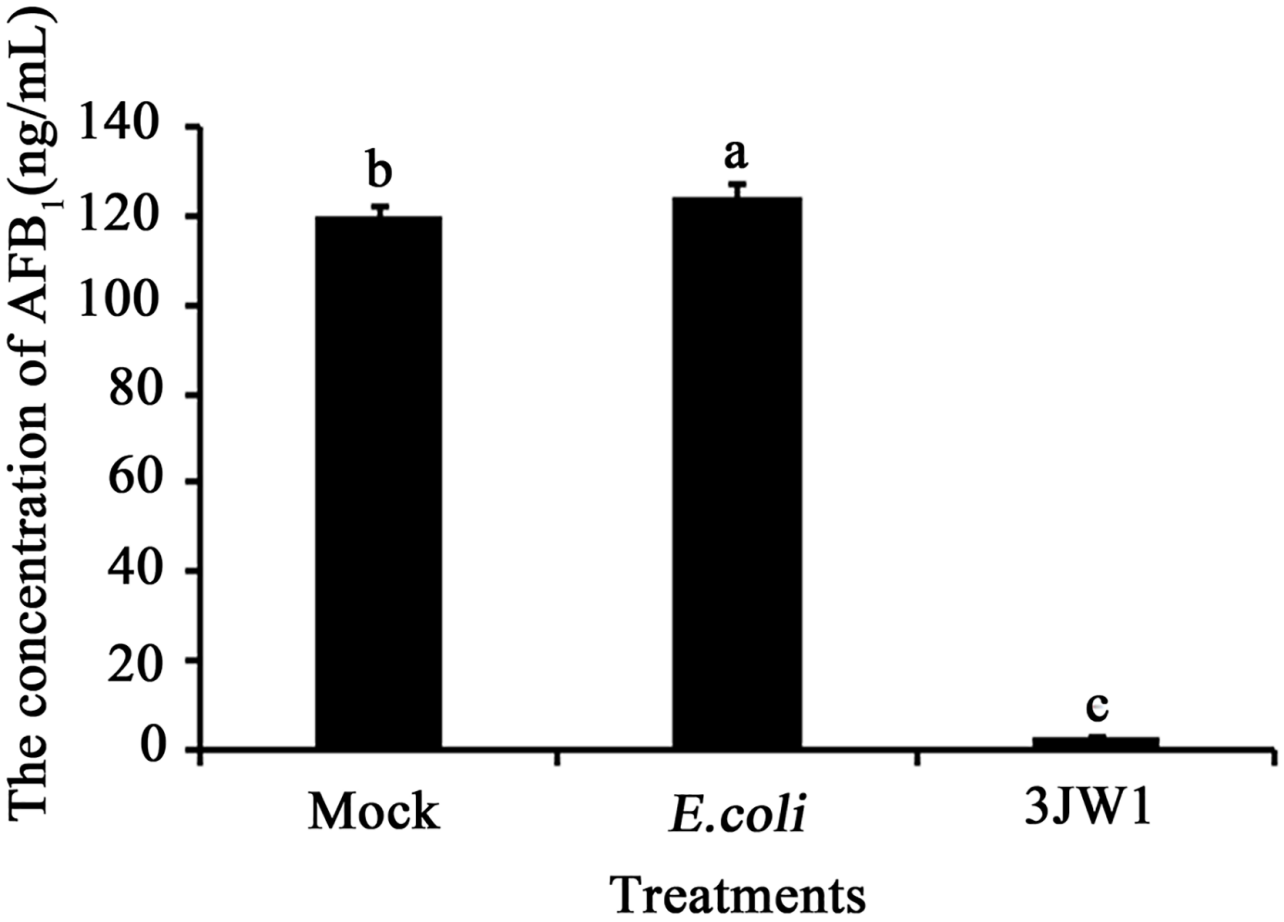
Effects of *Pseudomonas fluorescens* strain 3JW1 on AFB_1_ levels in PDB culture. *Aspergillus flavus* was mixed with *P*. *fluorescens* strain 3JW1 in a 100-ml flask containing 15 ml of PDB. The final concentrations of *A*. *flavus* and *P*. *fluorescens* strain 3JW1 in PDB were 5×10^5^ spores/ml and 1×10^7^ CFU/ml, respectively. A mock control with PDB alone and another control inoculated with 1×10^7^ CFU/ml *E*. *coli* TOP10F′ were used. After 4 d (96 h) of incubation with constant shaking (200 r/min, 28°C), the medium filtrate was collected (except mycelium), and the amount of AFB_1_ was determined by IAC-HPLC. This study was repeated 3 times, each with 3 replications.

When *A*. *flavus* and other strains were cultured together in PDB, the results showed that *E*. *coli* in the culture had no effect on AFB_1_ production. However, cultures inoculated with biocontrol strain 3JW1 showed significant reductions in the amounts of AFB_1_ produced in PDB (Fig 1), with the inhibition rate reaching 97.8%. A previous study showed that *Lactobacillus plantarum* K35 could inhibit *A*. *flavus* growth and could reduce the amount of AFB_1_ produced by *A*. *flavus* by 69% at 8.8 mg/ml of the supernatant [28]. In comparison to this and other biocontrol agents used in earlier studies [30, 33] to suppress aflatoxin production (Table 1), this *P fluorescen* strain 3JW1 appears to have more potential as a new biocontrol agent in practical applications.

**Table 1:** Mechanisms of aflatoxin suppression by various biocontrol agents against *Aspergillus flavus*.

Strain	Inhibit growth of *A*. *flavus*	Degradation of AFB_1_	Inhibit toxin production of the first generation	Inhibit toxin production of the later generation
*Pseudomonas fluorescens* 3JW1	Y	Y	Y	Y
*Lactobacillus plantarum* K35	Y	--	Y	--
*Bacillus subtilis* UTBSP1	Y	Y	Y	--
*Bacillus cereus*	Y	--	Y	--
*Bacillus megaterium*	Y	--	Y	--

Note: Y means yes.

--means no results showed in the experiment.

#### Effects of *P*. *fluorescens* strain 3JW1 on AFB_1_ production in peanut medium

**Fig 2:**
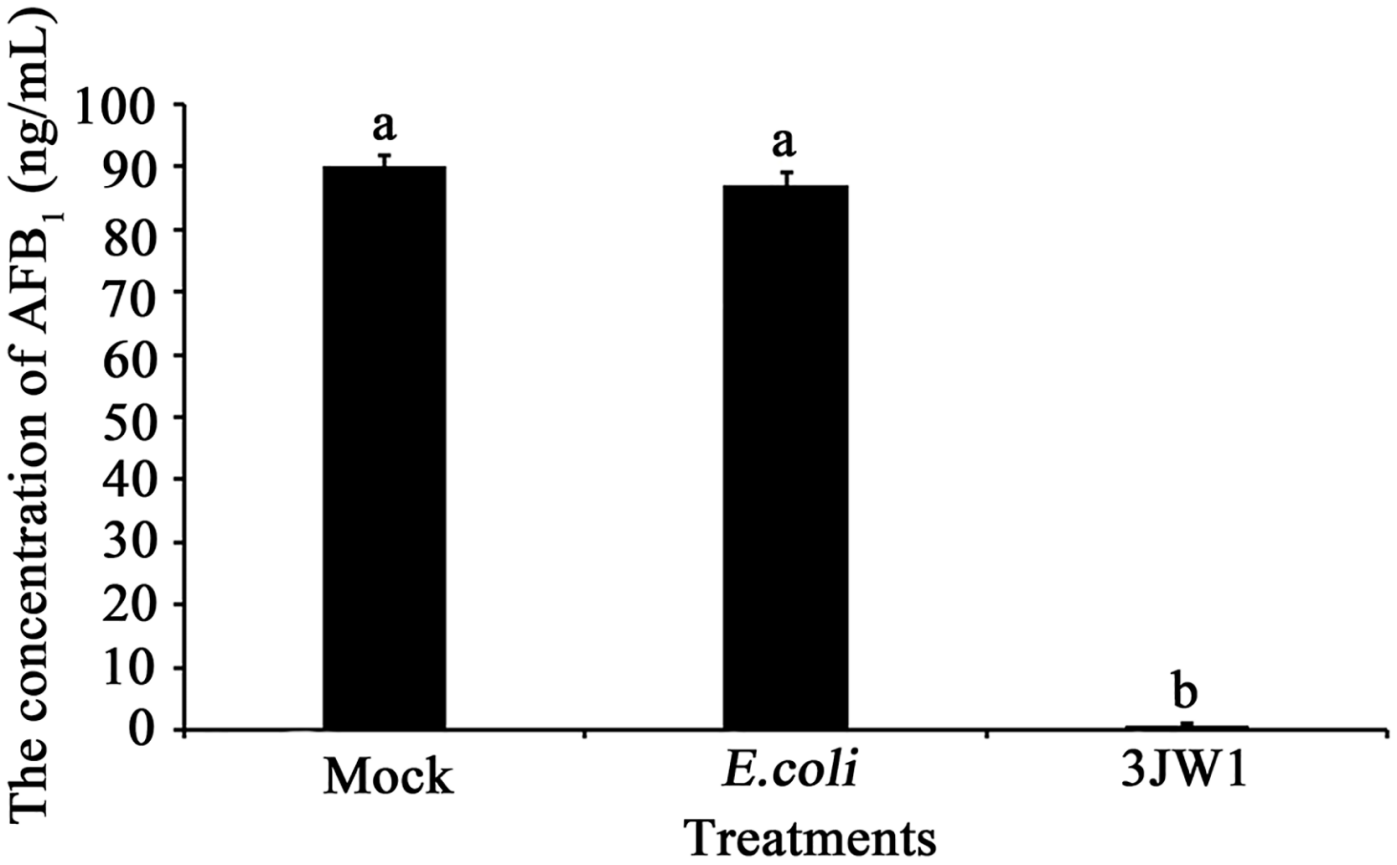
Effects of *Pseudomonas fluorescens* strain 3JW1 on AFB_1_ production in peanut medium. *Aspergillus flavus* was mixed with *P*. *fluorescens* strain 3JW1 in a 100-ml flask containing 15 ml of peanut medium. The final concentrations of *Aspergillus flavus* and *P*. *fluorescens* strain 3JW1 in PDB were 5×10^5^ spores/ml and 1×10^7^ CFU/ml, respectively. A mock control with PDB alone and another control inoculated with 1×10^7^ CFU/ml *E*. *coli* were used. After 96 h of incubation with constant shaking (200 r/min) at 28°C in an incubator shaker, the medium filtrates were collected, and the amount of AFB_1_ was determined by IAC-HPLC. This study was repeated 3 times, each with 3 replications.

Using the same method, when *A*. *flavus* and other strains were co-cultured in peanut medium, the result indicated that AFB_1_ was produced at high amounts in both controls. In contrast, AFB_1_ was barely detected in cultures that contained the biocontrol strain 3JW1 (Fig 2).

#### Efficacy of *P*. *fluorescens* strain 3JW1 on reducing AFB_1_ levels in peanuts

**Fig 3:**
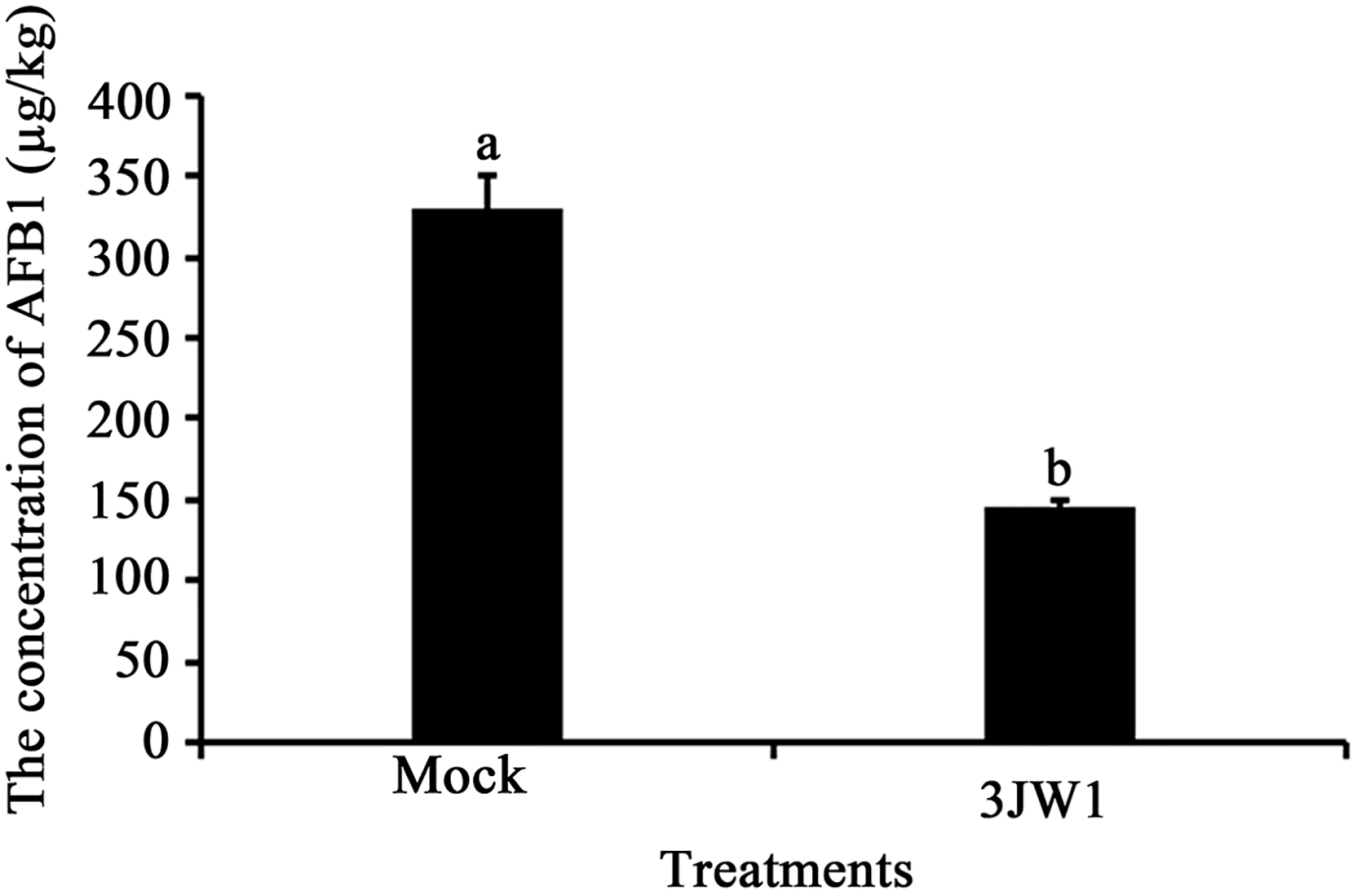
Effects of *Pseudomonas fluorescens* strain 3JW1 on AFB_1_ production in peanut kernels (μg/kg). Peanut kernels were surfaced-disinfected with 0.1% sodium hypochlorite, rinsed with sterile distilled water 3 times, air-dried in a petri dish. Tewenty peanut kernels were used in each assay. One ml of biocontrol strain 3JW1 at 1.0×10^7^ CFU/ml was added into petri dish, 4h later, 1 ml *A*. *flavus* at 1×10^7^ CFU/ml was added into petri dish. All petri dishes were placed in an artificial weather chamber (humidity 85%, 28°C). The amount of AFB_1_ was determined by IAC-HPLC 7 days later. Each treatment was replicated three times and this study was repeated four times.

To evaluate the efficacy of strain 3JW1 in suppressing aflatoxin contamination in practical application, peanut kernels with or without precoating them with the *P*. *fluorescens* strain 3JW1 were inoculated with *A*. *flavus*. The result indicated that strain 3JW1 significantly reduced the amount of aflatoxin contamination in peanut kernels (Fig 3). The aflatoxin levels in the treated kernels were reduced by 55.8% on the average or by ~183 micrograms AFB_1_ per kilogram peanut kernels (183 μg/kg) compared with the controls. The data presented herein demonstrated for the first time that *Pseudomonas fluorescens* exhibited inhibitory effects on the amounts of AFB_1_ produced by *A*. *flavus* on peanut kernels. In addition to *Lactobacillus plantarum* K35 [28], previous reports found that *Bacillus cereus* and *Bacillus megaterium* could control kernel rot in peanut caused by *A*. *flavus* [30, 33]. Reddy *et al*. reported that *Pseudomonas fluorescens* treatment could lead to a 62.6% reduction of AFB_1_ in sorghum grains [34].

### Effects of *P*. *fluorescens* strain 3JW1 on the growth of *A*. *flavus*

**Fig 4:**
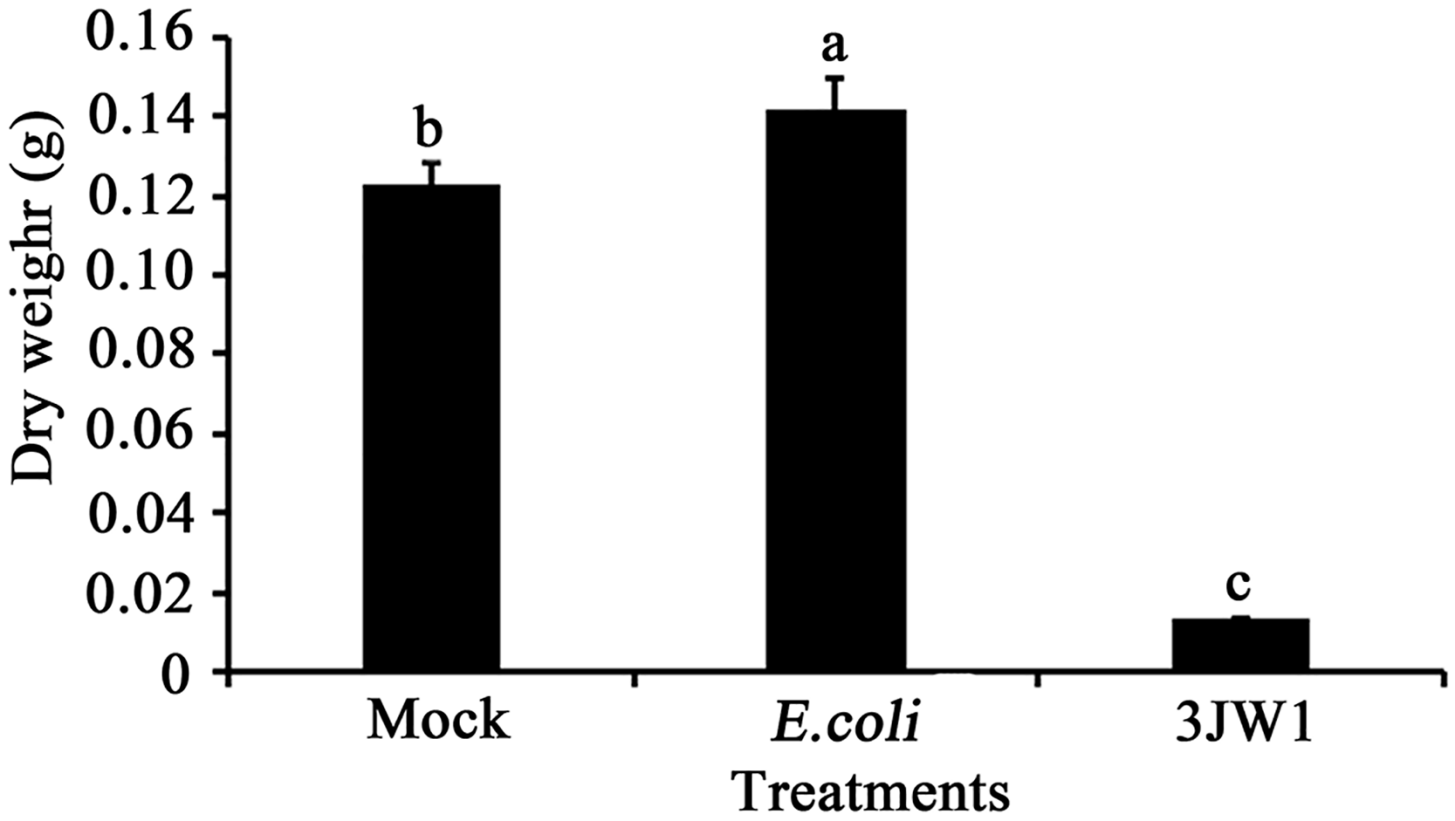
Effects of *Pseudomonas fluorescens* strain 3JW1 on the mycelial dry weight of *Aspergillus flavus* 73. *Aspergillus flavus* was mixed with *P*. *fluorescens* strain 3JW1 in a 100-ml flask containing 15 ml of PDB. The final concentrations of *A*. *flavus* and strain 3JW1 in PDB were 5×10^5^ spores/ml and 1×10^7^ CFU/ml, respectively. A mock control with PDB alone and another control inoculated with 1×10^7^ CFU/ml *E*. *coli* were used. After 4 d (96 h) of incubation with constant shaking (200 r/min, 28°C), the mycelium were collected, then washing and drying at 80°C for 24 h. The mycelium dry weight obtained was recorded. This study was repeated 3 times, each with 3 replications.

*A*. *flavus* (5×10^5^ spores/ml) and strain 3JW1 (1×10^7^ CFU/ml) were co-cultured in PDB to determine whether the biocontrol strain could inhibit fungal growth. A mock control with *A*. *flavus* in PDB alone and another control inoculated with 1×10^7^ CFU/ml *E*. *coli* were used. Mycelia of *A*. *flavus* were collected, and the mycelium dry weights were compared. The result (Fig 4) showed that comparing to both mock control treatment and *E*. *coli* treatment, strain 3JW1 significantly suppressed mycelial growth (over 80%) of *A*. *Flavus*.

### Aflatoxin production by *A*. *flavus* recovered from the *P*. *fluorescens* strain 3JW1-treated medium

**Fig 5:**
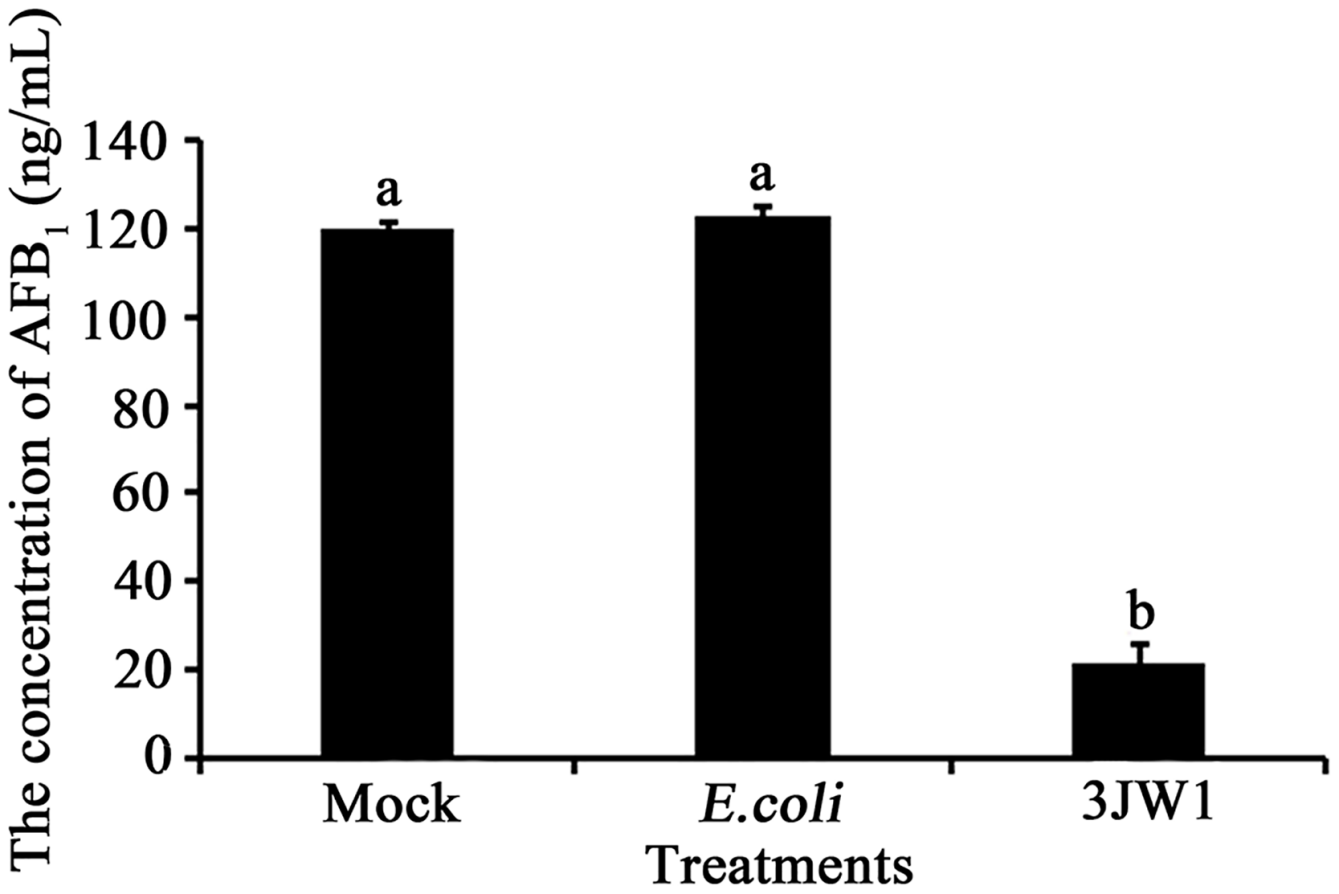
Production of AFB_1_ in the first sequential sub-culturing of *A*. *flavus* on PDB medium. One hundred microliter of *A*. *flavus* and strain 3JW1 mixed culture was collected at the end of 96 h co-incubation on PDB, then plated on petri dishes containing PDA medium. After 4 days, a single colony of *A*. *flavus* was transferred onto a new PDA plate. Conidia of *A*. *flavus* on the new plate were collected 14 days later, and were used to inoculate into 15 ml of PDB in a 100-ml flask at a final concentration of 5×10^5^ spores/ml. After 96 h of constantly shaking (200 r/min) the above culture at 28°C in an incubator shaker, the medium filtrate was collected and the amount of AFB_1_ was determined by IAC-HPLC. This study was conducted 3 times and each treatment was replicated three times. The initial conidial concentration of *A*. *flavus* in the PDB medium was 5×10^5^ spores/ml.

The above result showed the biocontrol strain 3JW1 reduced aflatoxin accumulation, however, it was not clear whether it resulted from inhibiting aflatoxin biosynthesis or degrading the synthesized aflatoxin. Therefore, the *A*. *flavus* strain 73 was recovered again from the medium co-cultured with strain 3JW1, and its aflatoxin synthesis ability was examined in PDB medium. The result (Fig 5) showed the aflatoxin production of *A*. *flavus* was still inhibited after the first sequential sub-culturing on PDB medium, which indicated that the biocontrol strain had the ability to inhibit aflatoxin synthesis of *A*. *Flavus*.

### Effects of *P*. *fluorescens* strain 3JW1 on AFB_1_ degradation

To determine whether strain 3JW1 could also degrade aflatoxins, AFB_1_ was mixed with the biocontrol strain 3JW1, and the final amounts of AFB_1_ were compared to controls after 4 days. Compared with the controls, strain 3JW1 showed the ability to degrade AFB_1,_ with the degradation rate reaching 88.3% in 4 days. In contrast, *E*. *coli* showed no ability to degrade AFB_1_. Previous study reported that AFB_1_ could be degraded into various other compounds (AFD_1_, AFD_2_, and AFD_3_) by *Pseudomonas putida* [35]. *Bacillus subtilis* UTBSP1 isolated from pistachio nuts from Iran can also degrade AFB_1_ [36].

In conclusion, through the series of experiments described above, the results showed that the *Pseudomonas fluorescens* strain 3JW1 could effectively reduce aflatoxin contamination on peanut kernels by not only suppress fungal growth and aflatoxin biosynthesis, but also breaking down the synthesized aflatoxin. As the field-isolated 3JW1 is relatively harmless, our findings suggest that it has great potential applications in both preventing pre-harvest aflatoxin contamination and degrading produced aflatoxins in the post-harvest agro-products.
